# A myoelectric digital twin for fast and realistic modelling in deep learning

**DOI:** 10.1038/s41467-023-37238-w

**Published:** 2023-03-23

**Authors:** Kostiantyn Maksymenko, Alexander Kenneth Clarke, Irene Mendez Guerra, Samuel Deslauriers-Gauthier, Dario Farina

**Affiliations:** 1 Neurodec, Sophia Antipolis, France; 2 Department of Bioengineering, Imperial College London, London, UK; 3 Centre Inria d’Université Côte d’Azur, Valbonne, France

**Keywords:** Electrical and electronic engineering, Computational biophysics

## Abstract

Muscle electrophysiology has emerged as a powerful tool to drive human machine interfaces, with many new recent applications outside the traditional clinical domains, such as robotics and virtual reality. However, more sophisticated, functional, and robust decoding algorithms are required to meet the fine control requirements of these applications. Deep learning has shown high potential in meeting these demands, but requires a large amount of high-quality annotated data, which is expensive and time-consuming to acquire. Data augmentation using simulations, a strategy applied in other deep learning applications, has never been attempted in electromyography due to the absence of computationally efficient models. We introduce a concept of Myoelectric Digital Twin - highly realistic and fast computational model tailored for the training of deep learning algorithms. It enables simulation of arbitrary large and perfectly annotated datasets of realistic electromyography signals, allowing new approaches to muscular signal decoding, accelerating the development of human-machine interfaces.

## Abstract (web summary)

Muscle electrophysiology is a promising tool for human-machine approaches in medicine and beyond clinical applications. The authors propose here a model simulating electric signals produced during human movements and apply this data for training of deep learning algorithms.

## Introduction

Biosignals have been classically used for studying the underlying physiology, for clinical diagnostics, and for monitoring. More recently, they have also been used for interfacing humans with external devices. For example, signals measured at the surface of the skin from skeletal muscle electrical activity, i.e. surface electromyography (sEMG), are used for the control of bionic limbs^1^. In this application, the recorded electrical signals are converted into motion commands using machine learning^2–4^. In recent years, with the development of deep-learning-based methods as well as wearable and cost-effective recording devices, there has been increased interest in using muscular signals as a basis for human-machine interfaces^5,6^. The potential applications go well beyond the traditional clinical domains of prostheses and orthoses and range from robotic control to gaming and virtual reality^7^.

To be useful in real-world applications, particularly for the mass market, EMG-based muscle-computer interfaces need to be highly intuitive and functional. They also need to work for the vast majority of subjects with little or no user-specific calibration, regardless of their anatomy and physiology. Traditional machine learning techniques might work well for the relatively simple task of hand gesture classification, but they require training on a specific subject and potential regular recalibrations^1,7^. This is a major bottleneck for mass market applications, where obtaining subject-specific data is not possible. Deep learning methods could tackle these problems^8^, but large, annotated, and high-quality datasets are required. To consider deep learning, training data needs to be recorded for different subjects, at different times, with high variability in electrode configurations and experimental paradigms. In addition, it is challenging and in some cases impossible to properly describe the underlying physiological or neural parameters (e.g. individual muscle forces, fibre physiological parameters, motor neuron impulse timings), which are crucial for the correct annotation of data samples. As a result, acquiring experimental EMG data in sufficient quantity and quality is not only expensive and time-consuming, but in many cases not possible.

Data augmentation via simulation is an alternative approach to lengthy data acquisitions, and indeed, augmentation techniques have been recently introduced for electrophysiological signals^9–12^. However, most of these augmentation methods use black-box models, which aim to capture essential features of the signal without relating them to the underlying physiology^13^. Thus, the ground truth for most of the crucial parameters is still unknown, greatly limiting the potential use cases of such approaches. More sophisticated biophysical modelling methods are based on solving so-called forward equations (e.g., Poisson equation in the electrostatics case). However, this type of biophysical modelling has not been considered in the context of data augmentation for machine learning approaches. Indeed, state-of-the-art models are either not sufficiently realistic or not computationally efficient to produce suitable training data. For example, in the case of describing the generation of EMG signals, analytical models based on simple geometries of the tissues^14–18^ provide simulations that reflect the broad characteristics of the signals, but cannot be used to reproduce specific experimental conditions due to the overly simplified anatomy. The more realistic models of EMG generation based on numerical solutions of the Poisson equation with generic volume conductor shapes^19,20^ are currently limited by their prohibitive computational time.

**Fig. 1:**
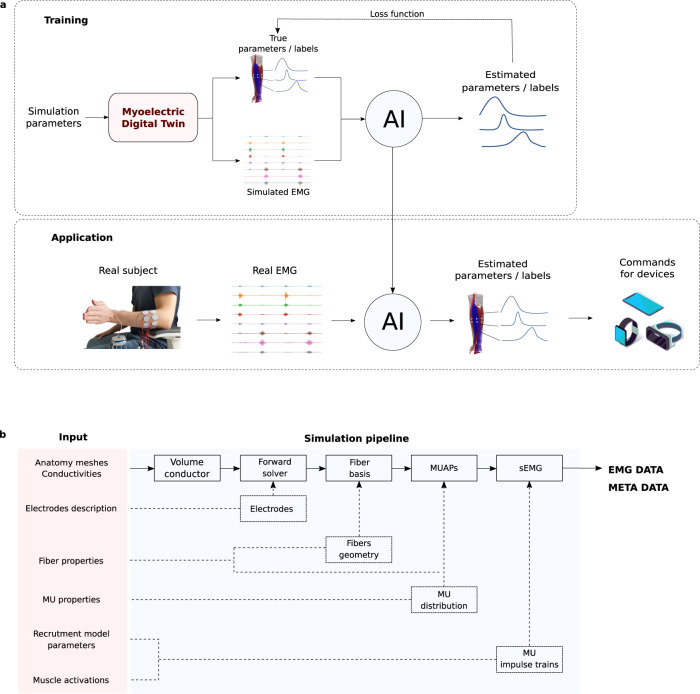
Simulation pipeline. **a** General strategy of using Myoelectric Digital Twin to train an artificial intelligence (AI), that then can be used to process real surface electromyography (sEMG) signals in real-world applications. **b** Schematic representation of the software’s input, main elements of the simulation pipeline and output. User can define a large set of simulation parameters describing anatomical and physiological properties of the tissues, geometry and montage of electrodes, fibres geometry and their physiological properties, motor unit (MU) distribution and their recruitment model, activation of individual muscles, etc. The main components of the simulation pipeline are described in details in ‘Methods’ section. For a given set of the parameters, the software outputs the resulting simulated EMG data as well as all the metadata of the simulation (e.g. individual motor unit action potentials (MUAPs)).

Here, we describe an EMG simulation method, based on the numerical solution of the forward equations suitable for deep learning data augmentation. It produces highly realistic EMG recordings, provides access to all underlying physiological parameters, and is significantly more computationally efficient, compared to the state-of-the-art methods. Although, there is no benchmark to evaluate and compare the exact performance of different simulation methods, our model takes minutes (or even seconds) to perform simulations that take hours with the state-of-the-art methods^20,21^. We achieved this gain in computational efficiency not by simply optimizing the code or using parallel computing, but by exploiting the mathematical and structural properties of the model and reformulating them theoretically. Our results show that it is possible to simulate EMG signals for anatomically accurate conductor geometries and multiple muscles with tens of thousands of muscle fibres in a few seconds. This realistic and fast model allows, for the first time, the generation of synthetic EMG data in sufficient quantity and quality to train deep learning algorithms. Figure 1a illustrates a general pipeline of using the Myoelectric Digital Twin to train EMG processing AI. At the training stage, the user can define a high variety of simulation parameters. The software generates a corresponding EMG dataset coupled with all the metadata that can potentially be used for annotation. The annotated data is then used to train a specific AI. This trained AI can then be used to process real EMG measurements and translate it into commands for real-world applications.

Note, that our model and the proposed pipeline are quite general and do not depend on a specific neural network architecture or the final application. As a concrete example, Fig. 1a shows an AI which is trained to reconstruct individual muscle forces from an EMG signal. In this case, the exact muscle forces provided as input to the simulation are used to annotate the simulated data. Notice that training such an algorithm with experimental EMG data would be impossible, because the ground truth of exact muscle forces is unavailable in real measurements. Once trained on synthetic data, the AI can be used on real subjects without calibration or fine-tuning. When a person makes some complex hand movements, the corresponding EMG signal is processed by the AI and the muscle activations are reconstructed in real time. This information can then be used to interfaces with different devices with high precision and complexity of the gestures.

This particular application is just an example. The same approach can be used for different tasks: categorical gesture classification, denoising, fatigue detection, and so on. Our approach is also agnostic to the exact neural network architecture. As an application scenario and a proof of concept, in this work, we demonstrate the use of this model for data augmentation by pre-training neural networks that decompose EMG into the underlying neural activity sent from the spinal cord to muscles^22^.

Note, that because large EMG dataset simulation was not possible before, the effect of using this data for AI training is not yet well studied. It includes all the potential advantages of using synthetic EMG data, as well as potential biases that it can introduce to the training. Our tool, however, opens doors for new research topics in this domain.

Our model is the only realistic and computationally efficient simulator targeted to AI training and approaching the concept of a Myoelectric Digital Twin. It allows generating arbitrary large datasets of realistic and personalized EMG signals, with high data variability and with a perfect annotation of diverse hidden parameters. As a result, our model may allow breakthrough approaches in AI-based EMG signal processing and decoding.

## Results

### Software structure and user interface

Our Myoelectric Digital Twin is a cloud-based software with a Python API which allows users a simple yet flexible way to define various simulation parameters and to control the simulation pipeline. User can define subject’s anatomy (by providing surfaces of the muscles, bones, fat and skin), different tissue conductivities, the electrode configuration (electrode locations, sizes, shapes), individual fibre properties (location of neuromuscular junctions, tendon lengths, action potential propagation velocities), motor unit and recruitment model parameters, as well as the activations of each muscle, etc.

Our software is not limited to individual muscle activations. Instead, it allows users to define arbitrary activation (% of MVC as a function of time) for each muscle of the model (e.g. a forearm) simultaneously. The corresponding EMG signal is a superposition of contributions of each individual muscle. Figure 1b shows a schematic representation of the user’s input, main simulation modules and their interaction. Each step of the simulation pipeline depends on the output of the previous module and on a specific subset of the input parameters. This architecture, dictated by the mathematical properties of the model, allows efficient management of pre-computed data (i.e. changing fibre properties does not require recomputing the forward solver). Moreover, users can provide their own data as an input for each module instead of using automated tools of our software, if necessary (see Supplementary Movie for a video example of a simulation script).

### Biophysics

To allow the efficient simulation of a large quantity of highly realistic EMG recordings, we have developed a novel approach to solve the forward problem of the volume conductor in electrostatic conditions. Our approach is based on a hierarchical and flexible decomposition of the EMG simulation pipeline, which allows the reuse and optimisation of individual steps.

In this section, we give a general overview of the model and advantages that it provides. The details and all mathematical equations related to the model development are described in the ‘Methods’.

First, a realistic anatomy, described by bone, muscle, skin, and electrode surfaces, is discretized into a tetrahedral volume mesh. A conductivity tensor, anisotropic for muscles and isotropic elsewhere, is associated with each tetrahedral of the volume. Unlike the state-of-the-art approaches, which solve the quasi-static Maxwell’s equations for each fibre source and for each time instant, we solve them for a set of unit point sources located at each vertex of the mesh associated with the muscle tetrahedra, which are referred to as basis sources. This computation does not depend on the time variable, nor on the fibres and motor unit geometry and their physiological properties. Therefore, changing these parameters does not require recomputing the forward solutions.

Moreover, due to a rewriting of the equations involved using the so-called adjoint method, the solution is obtained by solving as many systems of equations as there are electrodes, rather than basis sources. Because the number of electrodes (≈10^2^) is typically much lower than the number of basis sources (≈10^5^), computational performance is substantially improved.

Second, using the same muscle surfaces used to describe the volume conductor, individual fibre geometries can be automatically generated, if this data is not available from other sources (e.g. from diffusion magnetic resonance imaging). Moreover, the fibres are grouped into motor units (MUs) following the state-of-the-art models for MU physiology. This step does not depend on the forward computations, and thus altering the related parameters and producing new simulation is highly efficient.

Third, the current source density propagating along the fibres is generated using a realistic intracellular action potential model. The contribution of individual fibres to the EMG recordings is obtained by discretizing each fibre into a set of points, integrating the current source density along its length, and projecting onto the sensor locations using the basis points computed in the first step. This approach effectively decouples the number of fibres and their discretization from the conductor model, allowing the simulator to handle tens of thousands of fibres per muscle. Again, changing the fibre parameters (end-plate location, action potential propagation velocity, tendons length, etc.) does not require recomputing the other blocks of the simulation.

Fourth, given a muscle activation profile, we use the size principle to recruit MUs and their associated fibres. This allows a simple and easily interpretable input to the simulation that can be used to simulate EMG recordings associated to specific muscle contractions and their movements.

As a result, our model is the first that allows the generation of highly realistic and arbitrarily large (because of its computational performance) datasets of simulated EMG signals that can be used for AI training.

### The simulator reproduces analytical solutions

**Fig. 2:**
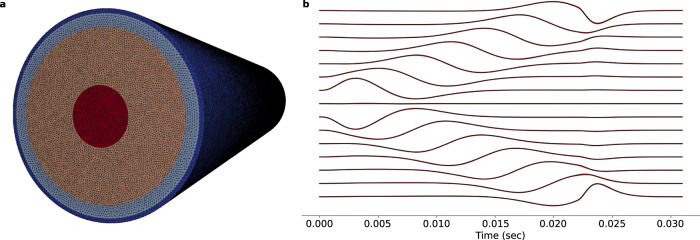
Comparison of the numerical and analytical solutions. **a** Tetrahedral mesh of the four layer cylindrical volume conductor. **b** Analytical^23^ (red) and numerical (black) EMG signals for a differential array electrode montage. The depth of the source fibre in this example is 1 mm from the muscle surface.

To produce realistic EMG data, the simulator leverages a flexible representation of the underlying anatomy and physiology. This flexibility does not only allow the use of realistic and personalized models, but also permits reproducing simple conductor geometry used in analytical solutions. We compare our numerical solution with its analytical counterpart for a cylindrical volume conductor geometry^23^. Taking into account the complexity of the model and the number of approximations used in computing the numerical solution, it is important to show that it reproduces the analytical solution with low error. The normalized mean square error between the two solutions depended on the depth of the fibre and varied between 3% (1-mm depth from the muscle surface) and 5% (11-mm depth). Figure 2 illustrates the analytical and numerical solutions for a fibre depth of 1 mm from the muscle surface. Because of the low error, the two waveforms are almost indistinguishable. It is important to note that the two volume conductor models in this validation are not identical. The theoretical/analytical solution is computed for an infinitely long cylinder (repeated periodically when discretized), while the numerical solution uses a cylinder of a large (sufficiently longer than the fibre and the electrode array), yet finite length. Increasing the length of the cylinder did not significantly alter the error.

### The simulator generates realistic EMG data

**Fig. 3:**
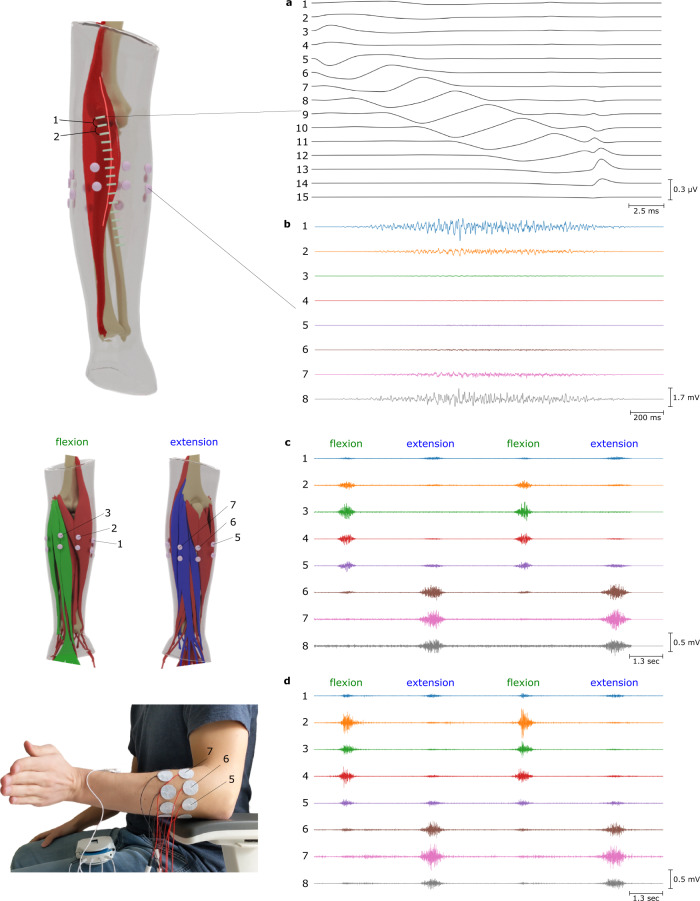
Simulation examples at multiple activation scales. **a** Single fibre activation in the Brachioradialis muscle measured by an electrode array with 15 differential channels. **b** 2-seconds long activation of the Brachioradialis muscle, reaching 100% of maximum voluntary contraction (MVC). 8 bipolar electrodes located around the forearm are simulated. **c** Simulation of wrist flexion and extension by activating the corresponding flexor and extensor muscles. **d** The experimental EMG signals of wrist flexion/extension.

To evaluate the performance of the simulator at multiple scales, we started by simulating EMG signals associated to a single fibre activation inside the brachioradialis muscle. The signal recorded by an array of 16 rectangular electrodes (15 differential channels) when a single fibre was active is shown in Fig. 3a. The volume conductor model is based on an anatomically accurate forearm geometry, which includes all the muscles, bones, fat and skin tissues.

Different distinctive features are present in the simulated signal that are also observed in experimental EMG signals^24^. In particular, electrodes of channel 4 are located on different sides of the neuromuscular junction (NMJ) and thus the respective signals cancel each other out. Channels 7–11 present propagating EMG components resulting from the fibre action potentials (AP) propagating from the NMJ to the tendons. Channels 2–6, as well as channels 12–15, contain non-propagating sEMG components, which are due to the AP generation at the NMJ and its extinction at the tendon (end-of-fibre effect), respectively.

A further example is a simulation of an excitation of a single muscle, illustrated in Fig. 3b. A simple excitation drive for the Brachioradialis muscle is simulated as gradually increasing from 0% to 100% of the maximum voluntary contraction and smoothly decreasing back to 0%. As described in the section ‘Computational performance’, 50,000 muscle fibres were realistically distributed into 200 motor units over the muscle volume and recruited according to the size principle^25^. The signal was simulated for 8 circular bipolar electrodes located around the forearm. In this example, the volume conductor effect becomes particularly visible with electrodes nearer to the active muscle having higher signal amplitudes. Notice that the electrodes record different signal waveforms as the muscle units are located at varying distances from the electrodes, weighting their contribution to the observed EMG signals. We also observe an increase of the signal amplitude with muscle excitation, an important feature of experimental EMG signals, which is a consequence of progressive motor unit recruitment and of an increase in the discharge rates of the active motor units.

Finally, we simulated sEMG signals from multiple muscle excitations, corresponding to the active wrist flexion and extension and passive wrist abduction against gravity. We used a simple muscle excitation model for three groups of muscles (flexors, extensors and abductors). More details about the experimental design are presented in the section ‘Details of realistic simulation examples’. Figure 3c and Fig. 3d clearly show the qualitative similarities in signal characteristics between experimental and simulated data. Our model was able to reproduce the different signal patterns during both flexion and extension. Beside the different activation across the electrodes during flexion and extension, the effect of wrist abduction is also visible in both datasets. Thus, channels 2, 3 and 7, 8 present a small signal activity during the whole duration of the simulation, and not only during flexion/extension peaks. Similar activity can also be seen in experimental data, with channels 2 and 7 being the most active.

**Fig. 4:**
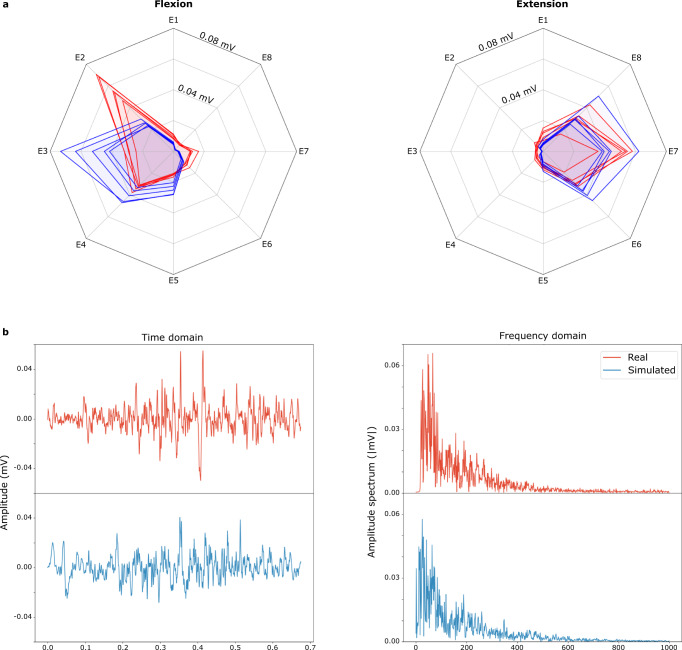
Signal features comparison. **a** Comparison of the root mean square values per electrode of experimental (red lines) and simulated (blue lines) surface electromyogram signals corresponding to 5 repetitions of hand flexion and extension. The electrode configuration is the same as shown in Fig. 3. **b** Comparison of experimental (red) and simulated (blue) single-channel surface electromyogram signals in the time and frequency domains. Here, some of the simulator parameters (e.g. fibres properties, motor unit recruitment model) have been optimized to match the experimental signals.

Figure 4a shows the comparison between the experimental and simulated signals for hand flexion and extension using root mean square (RMS) values per electrode. This is a pattern recognition feature which is commonly used in EMG. Most of the simulation parameters were fixed for default values, only the amplitudes of muscle activations were roughly chosen to match the real measurements (without a specific optimization). The simulated data reproduces well the RMS properties of the experimental signals. Yet, the match is not perfect, especially for the wrist flexion. This is, however, not surprising, because the model was not personalized, and simulation parameters were not optimized.

In addition to the analysis in the time domain, simulated data were compared against the experimental data in the frequency domain. Figure 4b illustrates an example of the measured and simulated single-channel sEMG. It has to be noted that the spectral characteristics of a signal strongly depends on multiple simulation parameters. In this example, we ran several hundreds of simulations by varying the simulation parameters in a realistic range and selected the set of parameters leading to the minimal spectral difference. This approach, which is a simple version of inverse modelling, was possible because of the high computational speed of the simulations.

### The simulator is fast

The computational performance of an EMG signal simulation depends on the model properties and the particular experimental setup. Consequently, there is no benchmark to evaluate and compare the performance of different simulation methods. The computational time magnitude of the state-of-the-art methods is, in the best cases, in the order of hours for a single simulation (with a fixed set of model parameter values, ≈50,000 fibres, 5 electrodes)^20,21^.

By exploiting the mathematical properties of the forward equations and the model architecture described in the previous sections, we were able to achieve a computational performance of the order of minutes per simulation. Because in our model, changing most of the simulation parameters does not require recomputing the whole model and reduces the computational time of new simulations to the order of seconds, if the volume conductor remains constant.

As a result, it becomes practically possible to simulate arbitrary large datasets of highly realistic EMG signals with high variability in the simulation parameters. Details on the computational time in several conditions are provided in Methods (section ‘Computational performance’).

The proposed model is also highly scalable for multiprocessing, and the current computational time can be further reduced by several orders of magnitude by implementing parallel computation on CPU and GPU.

### Realistic and fast EMG simulations open unique perspectives for deep learning

**Fig. 5:**
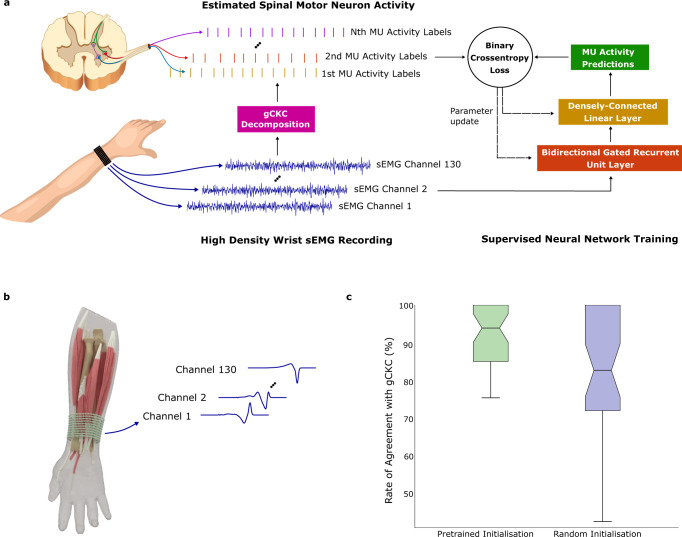
Results on the high-density EMG decomposition. **a** Decomposition of experimental high-density surface electromyography (HD-sEMG) recordings into underlying spinal motor neuron activities. The results obtained with the neural network (NN) were tested against the decomposition by a reference blind source separation method and manual editing by an expert operator. MU stands for motor unit. **b** Myoelectric digital twins were used to generate motor unit action potential (MUAP) templates for different muscles and different model parameters (tissue conductivities, fibre properties, tendon sizes, etc). 64 sets, each containing 5 simulated MUAPs for 130 electrodes, were used for pre-training. **c** Rate of agreement (%) between the neural network MU activity predictions and the decomposition algorithm on one second of wrist flexor HD-sEMG signal. Data is presented as median values over 39 motor units from nine participants +/− the interquartile range on the bounds of the box, with the lowest and highest values as the whiskers. Both outputs were converted to timestamps using a two class K-means clustering. The neural network using a gated recurrent unit (GRU) network that was pre-trained using simulated EMG signal significantly outperformed a GRU with random initialisation (two-tailed Wilcoxon signed-rank test, *Z* = 4.0, *p* = 0.00006, median difference 8.1 using Hodges-Lehmann estimator, 95% CI 3.4 to 13.3 using method of Walsh averages).

Here, we show a potential use of high volumes of simulated surface EMG data for deep learning, utilising the proposed model to generate data which can be used to pretrain neural networks. This methodology is used in other deep learning domains, such as the use of the ImageNet image database to pretrain object classifiers prior to adaptation to specific applications^26^. The selected application was that of a time series classifier, which took as an input unwhitened high-density surface EMG (HD-sEMG) signals recorded from the dominant wrist of nine participants and detected discrete MU activation events (Fig. 5a). Participants performed an isometric contraction at a constant force of 15% of the maximal voluntary contraction, as measured by a force cell and fed back to the user on a computer monitor. Only four seconds of data was used to optimise each network. The objective was to examine whether a neural network that was pre-trained using simulated sEMG and then fine-tuned on individual experimental data outperformed a neural network that used only a random initialisation, potentially compensating for low volumes of training data.

Specifically, the myoelectric digital twin simulation (Fig. 5b) was used to generate a large library of MUAP templates, which were then used to pretrain a gated recurrent unit (GRU) neural network architecture. GRU-based neural networks were selected because they have been shown to be effective at sEMG decomposition in past studies^27^, but pre-training is applicable to any neural network design, such as convolutional neural networks. The architecture of the GRU network was selected through grid search hyperparameter optimisation, and consisted of a single GRU cell with a hidden dimension of 1024. This was used to convert 130-channel HD-sEMG to a 1024-channel featurised signal, of which 20 samples were taken into a densely-connected linear layer for final prediction of MU activity for each time step.

The network was pre-trained using 320 simulation-generated MUAP templates. Training a classification network with an output dimension of 320 is very unstable when the target data is highly sparse and class-imbalanced, so instead a multitask framework was used. The task of training a classifier on 320 MUAPs was subdivided into 64 individual tasks, each predicting 5 MUAPs. The value of the GRU featurisation layer parameters were used and updated simultaneously for all 64 tasks, but each task used its own final linear layer with output dimension of 5. In this way, the GRU-layer could be pre-trained to find an efficient featurisation, which could then be used as a starting point for fine-tuning on the final prediction task with experimental wrist sEMG signals (see ‘Details of deep learning experiment’ for details).

The simulation pre-trained network outperformed random initialisation in decomposition accuracy when compared to the original decomposition as measured by the rate of agreement (RoA) metric calculated on a one second segment of data^28^ (Fig. 5c). The RoA metric efficiently incorporates false positive and false negative into a single metric of accuracy relevant to the decomposition of discrete events in time series data. The median (IQR) RoA of the pre-trained network was 93.8% (84.8 to 100.0), compared to 82.4% (71.6 to 100.0) in the random initialisation network, with a median difference of 8.1 (Hodges-Lehmann estimator, 95% CI 3.4 to 13.3 using method of Walsh averages), significant according to the two-tailed Wilcoxon signed-rank test (*p* = 0.00006, *Z* = 4.0). Of the 39 decoded motor units, 22 had improved RoAs with pre-training and one had a worse RoA, with the remaining 16 showing no change, generally because the initial RoA was already 100% without pre-training (these are large-amplitude MUAPs that are simple to detect). The pre-trained network had a much lower variance in the accuracy of predictions on the test sets than random initialisation, quickly optimising to a model effective for generalisation to new signals.

When units were subdivided into those from female (18 units) and male (21 units) subjects, both subsamples also showed a significant difference. For female subjects the median difference between trained and random initialisation was 9.1 (Hodges-Lehmann estimator, 95% CI 0.0 to 20.3 using method of Walsh averages) and significant (two-tailed Wilcoxon signed-rank, *p* = 0.00694, *Z* = 2.7). For male subjects the median difference between trained and random initialisation was 5.7 (Hodges-Lehmann estimator, 95% CI 1.6 to 9.7 using method of Walsh averages) and significant (two-tailed Wilcoxon signed-rank, *p* = 0.00064, *Z* = 3.4).

## Discussion

We have proposed an efficient computational approach to highly realistic surface EMG modelling. The method provides the solution to the generation of EMG signals from anatomically accurate volume conductor properties and number of muscle fibres, within limited computational time compatible with real-time signal generation. The proposed model is the only available EMG simulator with realistic description of the volume conductor and optimized for such computational efficiency. The main value of the model is that it opens perspectives for using simulated sEMG for data augmentation in the deep learning framework, something that was never done before using state-of-the-art simulation methods.

The computational efficiency in the volume conductor solution has been recognized as an important component of EMG modelling, and some attempts to decrease the computational time in EMG simulations have been described. For example, the approaches developed by Dimitrov and Dimitrova^29^ and Farina et al.^23,30^ substantially decreased the computational time in analytical EMG modelling for simple volume conductor geometries. These models provide simulations which reflect the broad characteristics of EMG signals, but cannot be anatomically accurate because of the restrictions on the volume conductor and fibre source geometry. Realistic models using numerical solutions have also been recently proposed. The previous most complete and efficient model has been proposed by Pereira Botelho et al.^20^. These authors have used an anatomically accurate model to simulate EMG signals generated during index finger flexion and abduction. They gained computational speed by using the principle of reciprocity. In fact, one part of our calculations also includes the adjoint method, which is an algebraic representation of this principle. By using reciprocity, Pereira Botelho et al.^20^ reported a computational time of 1 h for simulating the activation of nearly 15,500 fibres for 5 electrodes. This time, however, remains impractical for simulating arbitrary large datasets for a variety of parameter values. The model we proposed in this paper significantly surpasses the computational efficiency reported in ref. ^20^. We achieved it by efficiently exploiting mathematical properties of the forward equations, in particular by introducing the concept of basis points and by separating model parameters and variables into independent computational blocks. Our approach does not only reduce the computational time for a full simulation, but also allows us to scale the solution, so that new solutions for the same volume conductor can be obtained without recomputing the volume conductor transformation. In this way, the generation of EMG signals within the same volume conductor, but varying all other simulation parameters, can be performed in even shorter time. Complex EMG signals from tens of thousands of muscle fibres located in multiple muscles, can be generated (and regenerated with different parameter values) in a computational time of the order of seconds.

Some limitations remain in the current state of the presented model. It does not include some sources of variability that are present in experimental EMG signals and strongly impact their processing and analysis. For example, the model does not include advanced noise and artefacts descriptions, biomechanical modelling of the musculoskeletal system, and non-stationary volume conductor properties and fibre geometry.

Also, the automated modelling of muscle forces (or % MVC) for specific movements is out of the scope of this work. We recognize, however, the value of such a tool, and we see two potential ways to approach this task. First, by integrating our myoelectrical model with biomechanical modelling of the musculoskeletal system. This would allow users to define a movement by the dynamics of degrees of freedom (e.g. of a hand). Then, inverse dynamics could be used to estimate the corresponding muscle forces and % MVC, that can be used as an input to our current model. Another possibility is to estimate individual muscle activity from real EMG data. This would require an inverse problem solving algorithm. As a matter of fact, our Myoelectric Digital Twin is, practically, the only way to obtain labelled data for training such inverse algorithms, because the exact muscle forces of real subjects are not, in general, accessible.

While these aspects are beyond the scope of this paper, they are relevant features to include in future developments.

The advances presented in this work, together with the proposed future developments, naturally lead to the concept of a Myoelectric Digital Twin—a realistic, personalized, computationally-efficient model which generates EMG data in a quality and quantity sufficient not only to augment but to replace real data, with utility for AI training in the various real-world applications. Here, we have illustrated the potential of this approach by augmenting training data for deep neural networks, with the aim of identifying the discharge times of spinal motor neurons from surface EMG signal. By using the simulator to augment training (through a pre-training procedure), we showed an increase in the performance of the decomposition network when applied to experimental data, demonstrating a highly relevant use of the proposed approach for decreasing the need for experimental training data in human-machine interfacing applications.

## Methods

### Forward problem

The fibre extracellular potentials that are measured by EMG electrodes are generated by transmembrane currents. The properties of bioelectric currents and potential fields can be determined from solutions of the Maxwell’s equations, taking into account the electrical properties of biological tissues. Because of the relatively low frequencies of signal sources of biological origin, the quasi-static assumption can be applied^31,32^, so that the electric potential and the primary current sources are related by the following Poisson equation^31,33,34^ with Neumann boundary conditions:

1 $$\left\{\begin{array}{l}\nabla \cdot (\sigma \nabla \phi )=-I\quad \,{{\mbox{in}}}\;\Omega \quad \\ \sigma \frac{\partial \phi }{\partial {{{{{{{\bf{n}}}}}}}}}=\sigma \nabla \phi \cdot {{{{{{{\bf{n}}}}}}}}=0\quad \,{{\mbox{on}}}\,\partial \;\Omega \quad \end{array}\right.$$

where $\Omega \subset {{\mathbb{R}}}^{3}$ is a volume conductor domain of interest, ∂Ω its boundary with outward pointing normal unit vector **n**, *ϕ*(**r**)[*V*] is the electric potential, *I*(**r**)[*A*/*m*^3^] is the current source density (CSD), *σ*(**r**)[*S*/*m*] is a conductivity tensor. The second line of the equation (boundary condition) reflects the assumption that no current flows out of the domain of interest. In the context of EMG modelling, this implies that there is no current flow between the skin and air. The current source density *I*(**r**) is interpreted as the volume density of current entering or leaving the extracellular medium at position **r** ∈ Ω. A negative CSD corresponds to current leaving the extracellular medium (due to the fibre transmembrane currents) and is thus conventionally called a sink. Likewise, current entering the extracellular medium is called a source^35,36^.

**Fig. 6:**
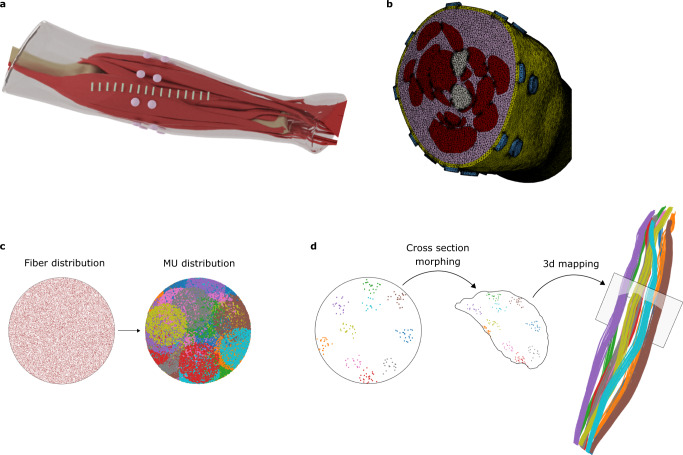
Model geometry. **a** Surface geometry of muscles, bones, subcutaneous tissue, skin and electrodes used for arm modelling (taken from BodyParts3D, The Database Center for Life Science (http://lifesciencedb.jp/bp3d/)). **b** Cross-section of the volume mesh generated from the arm surfaces. **c** Uniformly distributed fibres inside a unit circle are grouped into motor units (MUs) of different sizes, locations and territories. **d** Example of mapping of 10 small motor units from the circle into an arbitrary muscle by morphing the unit circle into the muscle cross section.

Equation (1) cannot be solved analytically for general volume conductor geometries, but several numerical methods can be used to approximate its solution. Here, we use the finite element method (FEM)^37^, which discretizes the volume conductor Ω as a tetrahedral mesh Ω_*t*_. Given this mesh, we use the Galerkin method to project the potential *ϕ* onto the space of piecewise affine functions defined on Ω_*t*_. Figure 6a and Fig. 6b illustrate an example of a realistic forearm model and corresponding discretized volume mesh, respectively.

This discretization process converts the continuous operator problem of Eq. (1) to a finite system of linear equations:

2 $$A{{{{{{{\bf{v}}}}}}}}={{{{{{{\bf{b}}}}}}}}$$

where *A* is a symmetric and sparse *n*_*v*_ × *n*_*v*_ matrix, *n*_*v*_ is the number of mesh vertices, ${{{{{{{\bf{v}}}}}}}}\in {{\mathbb{R}}}^{{n}_{v}}$ is a vector of potential values at mesh nodes, and ${{{{{{{\bf{b}}}}}}}}\in {{\mathbb{R}}}^{{n}_{v}}$ is a vector containing source information. Because the electric potential is defined up to a constant, the matrix *A* always has a one-dimensional null space. To obtain a unique solution to the system of Eq. (2), we constrain potentials **v** to have a zero sum.

In the context of EMG, we are not interested in finding electric potentials everywhere in the conductor, but only at the electrode locations. Let *S* be a selection matrix with a shape *n*_*e*_ × *n*_*v*_ which only selects the values at EMG electrode locations (*n*_*e*_ is the number of electrodes). Each row of *S* can be designed to select a single point location or to integrate over an area (e.g. the electrode-skin interface) depending on the location and number of its non-zero elements. Also, let **b**(**r**) correspond to a point source at location **r**. The resulting EMG signal is thus given by:

3 $${{{{{{{{\bf{v}}}}}}}}}_{{{{{{{{\bf{point}}}}}}}}}({{{{{{{\bf{r}}}}}}}})=S{A}^{-1}{{{{{{{\bf{b}}}}}}}}({{{{{{{\bf{r}}}}}}}}).$$

Let us analyse in more detail the structure of *A* and **b** from Eq. (2). Let {*w*^*i*^(**r**), *i* = 1…*n*_*v*_} be a set of *n*_*v*_*P*^1^ (piecewise linear) basis functions over the tetrahedral mesh Ω_*t*_. Note, that *w*^*i*^ is 1 at the *i*-th vertex of the mesh, is 0 at all other vertices and is linear at all tetrahedra adjacent to the *i*-th vertex. In this case, *A* and **b** have the following structure:

$${A}_{ij} 	={\int}_{{\Omega }_{t}}\sigma ({{{{{{{\bf{r}}}}}}}})\nabla {w}^{i}({{{{{{{\bf{r}}}}}}}})\nabla {w}^{j}({{{{{{{\bf{r}}}}}}}})d{{{{{{{\bf{r}}}}}}}}\\ {b}_{i}	={\int}_{{\Omega }_{t}}I({{{{{{{\bf{r}}}}}}}}){w}^{i}({{{{{{{\bf{r}}}}}}}})d{{{{{{{\bf{r}}}}}}}}.$$

First, let us notice that *A* is symmetric and, in general, a very large matrix which can be stored only because it is sparse. Indeed, the functions *w*^*i*^ have a compact support and their pairwise scalar product is non-zero only for neighbour functions. Since the pseudo-inverse (or the inverse) of a sparse matrix is usually not a sparse matrix, it is impractical to compute it because of the amount of memory needed to store it. Thus, iterative methods are typically used to solve the system of Eq. (2) for every given **b**.

Consider the case of $I({{{{{{{\bf{r}}}}}}}})=\delta ({{{{{{{\bf{r}}}}}}}}-\bar{{{{{{{{\bf{r}}}}}}}}})$ which corresponds to a unit point current source at a location $\bar{{{{{{{{\bf{r}}}}}}}}}$. Without loss of generality, we assume that this source is inside a tetrahedron formed by the vertices *i*_1_, …, *i*_4_ of the mesh. In this case, we obtain:

$${b}_{i}=\left\{\begin{array}{l}{\lambda }_{j},\;{{\mbox{if}}}\,i\in \{{i}_{1},\ldots,{i}_{4}\},\quad \\ 0,\,\hfill{{\mbox{otherwise}}}\,\quad \end{array}\right.$$

where {*λ*_*j*_, *j* = 1, …, 4} are the barycentric coordinates of the point $\bar{{{{{{{{\bf{r}}}}}}}}}$ inside the tetrahedron {*i*_1_, …, *i*_4_}. Applying this expression to Eq. (3), we obtain:

$${{{{{{{{\bf{v}}}}}}}}}_{{{{{{{{\bf{point}}}}}}}}}(\bar{{{{{{{{\bf{r}}}}}}}}})=S{A}^{-1}{{{{{{{\bf{b}}}}}}}}(\bar{{{{{{{{\bf{r}}}}}}}}})=S{A}^{-1}\overline{B}{{{{{{{\boldsymbol{\lambda }}}}}}}}.$$

where $\bar{B}$ is a *n*_*v*_ × 4 matrix with ${\overline{B}}_{{i}_{j},j}=1$ for *j* = 1, …, 4, and 0 otherwise. This implies that the solution of the system of Eq. (2) for any unit point source can be computed as a barycentric sum of solutions on the vertices of the corresponding tetrahedron. Therefore, it is sufficient to compute solutions of Eq. (2) for basis sources located on mesh vertices, to be able to evaluate a solution for any point inside this mesh efficiently. Let *n*_*s*_ be the number of such basis sources. For the most general case, when the source can be located anywhere inside the mesh and *n*_*s*_ = *n*_*v*_, let *B* be a *n*_*v*_ × *n*_*s*_ identity matrix. The objective is to compute basis solutions:

4 $${V}_{{{{{{{{\rm{basis}}}}}}}}}=S{A}^{-1}B$$

where *V*_basis_ is a *n*_*e*_ × *n*_*s*_ matrix, whose columns contain the solutions of Eq. (2) for a unit point source located at the corresponding mesh vertex. Hence, the potentials for any source location **r** is given by:

5 $${{{{{{{{\bf{v}}}}}}}}}_{{{{{{{{\bf{point}}}}}}}}}({{{{{{{\bf{r}}}}}}}})={V}_{{{{{{{{\rm{basis}}}}}}}}}{{{{{{{\boldsymbol{\lambda }}}}}}}}({{{{{{{\bf{r}}}}}}}})$$

where ${{{{{{{\boldsymbol{\lambda }}}}}}}}({{{{{{{\bf{r}}}}}}}})\in {{\mathbb{R}}}^{{n}_{s}}$ is a vector, whose four non-zero elements contain the barycentric coordinates of a point **r** inside a corresponding tetrahedron. Note, that one may restrict potential sources to be located inside specific subdomains of the whole mesh (which is the case for EMG). In this case, *n*_*s*_ corresponds to the number of vertices of these subdomains, and the matrix *B* is a submatrix of the identity matrix.

The most straightforward way to compute *V*_basis_ from Eq. (4) is to solve a problem of the form *A***x** = **b**_**i**_ for each column of the matrix *B*. It would thus require solving *n*_*s*_ systems of linear equations. For realistic conductor geometries, which have a large number of vertices, solving a single system may take up to a few minutes and solving *n*_*s*_ systems quickly becomes impractical. Therefore, we propose the use of the adjoint method^38^, which requires solving *n*_*e*_ systems only. In the context of EMG, the number of electrodes is usually significantly smaller than the number of vertices in the muscle subdomain meshes, i.e. *n*_*e*_ << *n*_*s*_. Let us define *K* = *SA*^−1^, which is a matrix of size *n*_*e*_ × *n*_*v*_. Because *A* is symmetric, and the inverse of a symmetric matrix is also symmetric, we can write *K*^*T*^ = *A*^−1^*S*^*T*^. Then, *K* can be found by solving the system:

6 $$A{K}^{T}={S}^{T}.$$

The matrix *S*^*T*^ has *n*_*e*_ columns and, thus, only *n*_*e*_ linear systems need to be solved to find *K*. The basis solutions can then be found as:

7 $${V}_{{{{{{{{\rm{basis}}}}}}}}}=KB.$$

### EMG signal of a single fibre activation

The action potential generated by the flow of ionic currents across the muscle fibre membrane is the source of excitation. For a given intracellular action potential (IAP) model *V*_*m*_(*z*), the transmembrane current source per unit length is proportional to the second derivative of *V*_*m*_(*z*), where *z* is a fibre arc length measured in mm. A general description of the current source density travelling at velocity *v* along the fibre with the origin at the neuromuscular junction at location *z*_0_ is^28,30,39^:

8 $$I(z,t)={\sigma }_{{{{{{{{\rm{in}}}}}}}}}\pi {r}^{2}\cdot \frac{\partial }{\partial z}\left[\psi (z-{z}_{0}-vt){w}_{{L}_{1}}\left(z-{z}_{0}-\frac{{L}_{1}}{2}\right)\\ -\psi (-z+{z}_{0}-vt){w}_{{L}_{2}}\left(z-{z}_{0}+\frac{{L}_{2}}{2}\right)\right]$$

where *z* ∈ [0, *L*] is a location along the fibre of length *L*, $\psi (z)=\frac{d}{dz}{V}_{m}(-z)$, *L*_1_ and *L*_2_ are the semi-lengths of the fibre from the end-plate to the right and to the left tendon, respectively, *σ*_in_ is the intracellular conductivity, and *r* is the fibre radius. We have chosen *w*_*L*_ to be a Tukey window, as proposed in ref. ^21^. The intracellular action potentials (IAP) ${V}_{m}\left[\frac{mV}{mm}\right]$ can be mathematically described in the space domain as proposed in ref. ^40^:

$${V}_{m}(z)=96{z}^{3}{e}^{-z}-90.$$

Let **r**(*z*) be a fibre geometry parametrized with respect to the fibre arc length *z*. Combining the transfer function of a point source in Eq. (3) with the fibre’s current density in Eq. (8), we obtain the equation for the EMG signal resulting from a single fibre activation:

9 $${{{{{{{{\bf{v}}}}}}}}}_{{{{{{{{\bf{fibre}}}}}}}}}(t)=\int{{{{{{{{\bf{v}}}}}}}}}_{{{{{{{{\bf{point}}}}}}}}}({{{{{{{\bf{r}}}}}}}}(z))\,I\,(z,t)dz.$$

This integral can be efficiently approximated by discretizing the fibre geometry into sufficiently dense spatial samples ${\{{{{{{{{\bf{r}}}}}}}}({z}_{i})\}}_{i}$ and assuming that **v**_**point**_(**r**(*z*)) is piecewise constant around these points. If we also rewrite Eq. (8) in a shorter form as $I(z,\,t)={\sigma }_{{{{{{{{\rm{in}}}}}}}}}\pi {r}^{2}\cdot \frac{\partial }{\partial z}F(z,\,t)$, Eq. (9) becomes:

10 $${{{{{{{{\bf{v}}}}}}}}}_{{{{{{{{\bf{fibre}}}}}}}}}(t)\,	 \approx \, \mathop{\sum}\limits_{i}{{{{{{{{\bf{v}}}}}}}}}_{{{{{{{{\bf{point}}}}}}}}}({{{{{{{\bf{r}}}}}}}}({z}_{i}))\int\nolimits_{{z}_{i}-{\Delta }_{i}}^{{z}_{i}+{\Delta }_{i}}I(z,t)dz \\ 	 \,=\mathop{\sum}\limits_{i}{{{{{{{{\bf{v}}}}}}}}}_{{{{{{{{\bf{point}}}}}}}}}({{{{{{{\bf{r}}}}}}}}({z}_{i}))\int\nolimits_{{z}_{i}-{\Delta }_{i}}^{{z}_{i}+{\Delta }_{i}}{\sigma }_{{{{{{{{\rm{in}}}}}}}}}\pi {r}^{2}\cdot \frac{\partial }{\partial z}F(z,t)dz \\ 	 \,={\sigma }_{{{{{{{{\rm{in}}}}}}}}}\pi {r}^{2}\mathop{\sum}\limits_{i}{{{{{{{{\bf{v}}}}}}}}}_{{{{{{{{\bf{point}}}}}}}}}({{{{{{{\bf{r}}}}}}}}({z}_{i}))\left(F({z}_{i}+{\Delta }_{i},t)-F({z}_{i}-{\Delta }_{i},t)\right).$$

Note, that **v**_**point**_(**r**(*z*_*i*_)) can be efficiently computed from Eq. (5). Moreover, once **v**_**point**_(**r**(*z*_*i*_)) are computed for all given fibres, we can change the parameters of the current source density (action potential waveform shape, propagation velocity, location of neuromuscular junction), and compute the corresponding EMG signal with Eq. (10) by only matrix multiplication complexity.

### Geometrical and physiological modelling of motor units

The motor unit action potential (MUAP) is the summation of the single fibre action potentials (APs) of the muscle fibres in the MU. Different types of MUs can be modelled^41,42^. Our approach consists in generating fibre and motor unit distributions inside a unit circle, and then projecting it into arbitrary 3D muscle geometry (Fig. 6d), using methods similar to those described in ref. ^43^. This provides a high level of control for the fibre and MU distribution parameters independently of a particular muscle geometry. A common way to simulate fibres and MUs is to start by defining MU positions, sizes and territories, and then simulate fibres inside these MUs according to their parameters^44,45^. We, however, propose another approach. First, we simulate uniformly distributed fibres inside a unit circle. Then, MU centres and their circular territories are generated and, finally, we associate each fibre to an MU. A fibre is associated to one of the MUs that contains it inside its territory with a probability proportional to the MU density (Fig. 6c). This approach has two main advantages. First, it guaranties (by construction) the uniform fibre distribution inside a circular muscle cross-section. Second, once fibres are generated and projected into a muscle geometry, different MU distributions can be generated very quickly, without regenerating fibres and recomputing transfer functions **v**_**point**_(**r**(*z*_*i*_)) for their nodes.

#### MU recruitment model

During muscle contraction, the MUs are recruited according to the size principle^25^. This can be simulated by associating a threshold of excitation to each MU, as described for example by Fuglevand et al.^46^. Linear or non-linear rate coding models can be used^46–48^.

The excitation rate as a function of time for each muscle is converted into the firing rates of the active MUs. Inter-discharge intervals are then generated with variability of the discharges around the mean firing interval^49^.

### Implementation remarks

The implementation of the main steps presented in the previous section can be summarized as follows. Once the matrices *S*, *A* and *B* are computed, the matrix *K* is determined using Eq. (6) by solving *n*_*e*_ linear systems. Then, Eq. (7) is used to find the solutions for *n*_*s*_ basis points, which is a fast matrix multiplication operation. For any given point source location **r**, we compute its barycentric coordinates in associated tetrahedron and apply Eq. (5) to get values of electrical potentials at electrode locations. Finally, for a given fibre geometry, the single fibre action potential as recorded by the EMG electrodes is computed using Eq. (9).

The results presented in this study are obtained using a Python implementation of the proposed strategy. Assembling the matrix *A* and solving the system (6) is delegated to the FEniCS computing platform^50,51^. The forearm geometry that is here representatively used as a conductor model is taken from the website of BodyParts3D, The Database Center for Life Science (http://lifesciencedb.jp/bp3d/). The volume mesh is generated from the surface meshes of the forearm tissues using the CGAL C++ library^52^.

### Computational performance

In this section, we report the computational time of the proposed model for a specific simulation case. The exact computational time values strongly depend on the implementation, experiment design, model parameters etc. The order of magnitude, however, stays the same. Note, that no multiprocessing tools were used in these computations. Each step, however, is highly scalable and can be efficiently distributed between parallel processes, which would significantly increase the performance. Computations for each muscle and fibre are independent and can be performed in parallel. Parallel computing would also apply to the electrodes in the general basis points computation.

The reduction of the time complexity allowed by our model can be generally expressed in the following way. Solving forward equations naively, i.e. for each fibre and each time sample separately, the corresponding complexity is in order of the product of corresponding parameters *O*(∏_*i*_*n*_*i*_), where *n*_*i*_ are respectively the number of mesh vertices, electrodes, fibres, time samples, etc. Our method decomposes this original product into a sum of smaller products of parameter subsets $O({\sum }_{j}{\prod }_{i\in {I}_{j}}{n}_{i})$. Each such sub-product corresponds to a specific step in the simulation pipeline. For example, computing the forward solver is dominated by solving Eq. (6) with the conjugate gradient method, which only depends on the number of electrodes and mesh vertices. Not only this decomposition reduces the overall time complexity, but also allows using pre-computed data for a new simulation if only a subset of parameters is changed.

For the purpose of demonstration, we simulated a 1-min-long, 100% maximum voluntary contraction (MVC) excitation of the Brachioradialis muscle with 50,000 individual fibres and 200 motor units. The mesh of the volume conductor contained 2.1M vertices, which formed 13M tetrahedra. 16 rectangular and 16 circular electrodes were included in the model. The sampling frequency of the simulated signals was 2000 Hz. Table 1 shows the computational time for each of the main steps in this simulation.

**Table 1:** Computational performance of each of the main steps of a raw EMG simulation

General forward solution	Fibres basis points	Fibres EMG response	MUAPs assembling	Raw sEMG assembling
7 min (13 s/elec)	2 min	30 s	0.8 s	2.6 s

General basis points computation refers to Eq. (7); fibre basis points are computed with Eq. (5); fibres EMG response is computed with Eq. (9).

An important property of our model is that each step depends only on the data produced by the previous steps. This property can be exploited to change some simulation parameters without recomputing every step of the simulation. For example, it is not necessary to recompute solutions for the fibre basis points if fibres geometry and conductor model stay the same and only the parameters related to the fibre properties (AP velocity, end-plate location, tendon sizes, etc.), MU distribution or recruitment model are modified. In this example, the total simulation time for this new set of parameters will only take approximately 30 s + 0.8 s + 2.6 s = 33.4 s.

A brief description of the main parameters required at each step follows. The full arm and electrode geometry as well as the tissue conductivities define the computation of general basis points. To compute fibres basis points solutions, the 3D geometry of the fibres is required. Computing the fibre EMG responses requires the shape of the intracellular AP waveforms, AP propagation velocity, sizes of tendon and active fibre parts, neuromuscular junction location, fibre diameter and intracellular conductivity, and sampling frequency. To compute the MUs action potentials, the MU distribution in the muscle, i.e. the association of fibres to each motor unit, need to be defined. In the proposed model, once the number of MUs, their sizes and territory areas are selected, the MU distribution is randomly generated. Finally, to synthesize the sEMG signal, the muscle excitation drives and recruitment model parameters (motor unit recruitment thresholds and firing rates) are required.

### Comparison with the cylindrical analytical solution

First, we compared our numerical solution with its analytical counterpart for a simple volume conductor geometry^23^. We used a four layer cylindrical model with layers corresponding to bone (*r* = 0.7 cm), muscle (*r* = 2 cm), fat (*r* = 2.3 cm) and skin (*r* = 2.4 cm) surfaces. 16 point electrodes were simulated on the skin surface directly above a fibre. The fibre was located at varying depths into the muscle tissue, in the range of 1–11 mm. Differential sEMG signals were simulated using the analytical and numerical solutions of the forward problem.

### Details of realistic simulation examples

For the single muscle excitation example, 50k muscle fibres were generated inside the muscle and distributed within 200 motor units. The size of MUs varied exponentially from 11 to 1150 fibres. The areas of MU territories varied from 10% to 50% of the muscle cross-sectional area. The muscle excitation drive was decomposed into MU impulse trains according to the size principle. In this example, the firing rate for each MU ranged from 8 Hz to 35 Hz and all MUs were recruited when an excitation level of 75% MVC was reached.

For the multiple muscles experiment, the flexor group included the Palmaris longus, Flexor carpi ulnaris (ulnar head), Flexor carpi ulnaris (humeral head), and Flexor carpi radialis muscles. The extensor group included the Extensor digitorum, Extensor carpi ulnaris, Extensor carpi radialis brevis, and Extensor carpi radialis longus muscles. During a wrist flexion, the muscles of the flexor group reached an excitation level of 50% MVC. During extension, the extensor group was activated with the same excitation level. Moreover, a small but constant excitation (7% MVC) of the abduction muscle group was added to simulate the wrist resistance against gravity. The abduction muscle group included the Flexor carpi radialis, Extensor carpi radialis brevis, and Extensor carpi radialis longus muscles. For each muscle, a number of muscle fibres between 32k and 78k was simulated, depending on the muscle cross-sectional area. Muscle fibres were distributed within motor units, whose number varied from 150 to 300 per muscle.

### Details of deep learning experiment

To evaluate the effect of using the simulation-pretrained network, an experimentally collected high-density surface electromyography (HD-sEMG) signal dataset was used, originally created to test low-force human-computer interaction with wrist-wearable interfaces^7^. The experimental protocol was designed in agreement with the Declaration of Helsinki and was approved by Imperial College London ethics committee (JRCO: 18IC4685). A balanced gender distribution (as self-reported) was prioritised during participants’ recruitment. Nine participants (4 females, 5 males, ages: 23–31) took part in the study after signing informed consent forms. Participants were not compensated for their involvement in the experiments. The participants performed 5-s isometric contractions of their dominant-hand index finger at 15% of maximal force, with sEMG activity measured using two flexible 5x13 electrode grids with 8-mm spacing placed on the circumference of the wrist, immediately proximal to the ulnar head. HD-sEMG signal was acquired using a Quattrocento amplifier (OT Bioelettronica) at 2048 Hz sampling frequency, while force profiles were sampled with a Phidget load cell at 10 Hz. A Matlab 2019b (The MathWorks, Inc) program was used to synchronyse both modalities. The HD-sEMG signal was then decomposed into motor neuron activity using convolutive blind source separation^53^. For the purpose of training and testing the supervised decomposition pipeline, motor neuron activity was accepted if it was present for at least 80% of the contraction window. For each participant the HD-sEMG signal and accompanying decomposed motor neuron activity (as a sparse binary matrix) was then split into a 4-s training window and a 1-s testing window.

A gated recurrent unit (GRU) neural network was used as the deep learning model due to previous studies showing good performance with this data type^27^. After hyperparameter optimisation by grid search, a minimally-parameterised model was found to perform optimally, likely due to the short length of the training data available. Input HD-sEMG signal was first encoded by a single layer GRU with a hidden dimension of 1024 in length^54^. To make a time instant prediction a densely-connected linear layer with sigmoid activation function took as an input a moving 20 sample-wide window from the GRU output, centred on the time instant of interest. Predicted activity was converted to spike timestamps using a two-class K-means clustering algorithm. Binary cross entropy was used as the loss function and Adam with weight decay used as the optimising algorithm^55^.

To improve model generalisation an early-stopping framework was used, based on 10% of the training data retained as a validation set. Training, validation and test data was z-score standardised using the mean and standard deviation calculated from the training set. During training the input signal was augmented with noise of standard normal distribution. To account for the high sparsity of the output matrix, samples containing motor neurons were artificially oversampled, with each input batch of 512 time instants containing at least 20% motor neuron activation. All machine learning was implemented using the PyTorch library in python. Final performance was assessed using the rate of agreement metric (RoA).

**Fig. 7:**
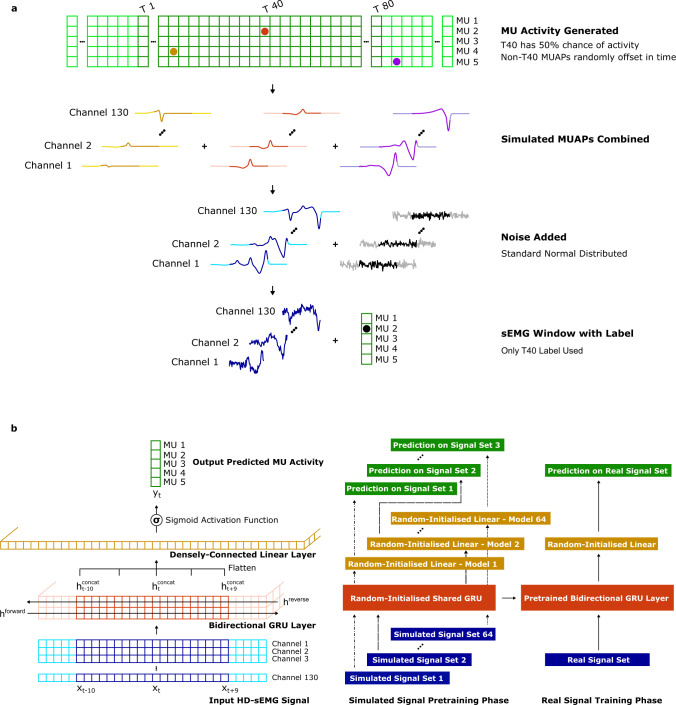
Neural network training pipeline. **a** Methodology used to build windows from the simulated MUAP template set for the pre-training phase. Each simulated template was 160 samples wide at a 2048 Hz sampling rate and with 130 channels. First, either a MUAP template was placed in the centre of the window or it was left empty at a 50% probability. Then MUAP templates from other MU classes were added to the window at a random offset to generate superpositions. Finally, standard normal distributed noise was added to the window, with the central 80 samples then paired with the label for supervised learning. **b** The neural network architecture and pre-training methodology used to improve the performance of a deep learning-based HD-sEMG decomposition algorithm. The neural network consists of a single gated recurrent unit layer, with predictions made using a 20-sample wide window of the hidden vector output, which is flattened before being passed to a sigmoid-activated densely-connected linear layer. In the pre-training phase a multitask learning regimen is used to optimise the parameters of the gated recurrent unit using the simulated sEMG. This pre-trained layer can then be used to improve the optimisation performance on real sEMG data.

The optimised architecture of the GRU network was used for pre-training, which was conducted using multitask learning in a hard parameter sharing paradigm^56^. Four digital twins were created for simulation using different model parameters (tissue conductivities, MU distribution, fibre properties, etc.), with the generated motor unit activation (MUAP) templates from flexor digitorum profundus and superficialis used to create 64 sets, each containing 5 MUAPs. Each set was used to generate windows of signal with a range of MUAP superpositions (Fig. 7a). In signal windows with motor neuron activity a MUAP template was placed in the centre of the window, before being additively superimposed with a random number of MUAP templates from other motor units at random time offsets. In windows without activity no template was placed in the centre of the window. During multitask learning training, the same GRU layer (and parameters) were shared between the 64 recordings, but each recording had its own output layer, operating on a 20 sample-wide window as in the experimental recordings (Fig. 7b). In this way the GRU layer was trained to act as a more general feature extractor, while the individual linear output layers made class predictions specific to each recording. Training again used noise augmentation, binary cross-entropy and Adam with weight decay.

To use the simulation-pre-trained network in the experimental data the GRU parameters from the pre-trained network were used, while the linear output layer used a normal random initialisation. This was the compared to a normal random initialisation of both the GRU and output layer. In both instances the network was trained using the methodology specified above, with the only difference being whether the GRU layer was simulation-pre-trained or not.

### Reporting summary

Further information on research design is available in the Nature Portfolio Reporting Summary linked to this article.

## Supplementary information

Description of Additional Supplementary Files

Supplementary Video 1

Reporting Summary

## Data Availability

Surface geometry of muscles, bones, subcutaneous tissue and skin data used for arm modelling were taken from BodyParts3D, The Database Center for Life Science (http://lifesciencedb.jp/bp3d/). The simulated MUAPs data that was used to train the neural network in the deep learning experiment have been deposited in the Science Data Bank database under 10.57760/sciencedb.07548 and CC BY-NC-SA 4.0 licence. The raw experimental data that was used in the deep learning experiment has also been deposited in the Science data Bank database under 10.57760/sciencedb.07586 and CC BY-NC 4.0 licence.

## Code availability

The EMG simulation software, the code is proprietary and is not publicly available. To request the access to the simulation software, please contact kostiantyn.maksymenko@neurodec.ai.

## References

[1] Farina, D. et al. Toward higher-performance bionic limbs for wider clinical use. *Nat. Biomed. Eng.*10.1038/s41551-021-00732-x (2021). 10.1038/s41551-021-00732-x 34059810

[2] Farina D (2014). The extraction of neural information from the surface EMG for the control of upper-limb prostheses: Emerging avenues and challenges. IEEE Trans. Neural Syst. Rehabilitation Eng..

[3] Farina D (2017). Man/machine interface based on the discharge timings of spinal motor neurons after targeted muscle reinnervation. Nat. Biomed. Eng..

[4] Zhuang KZ (2019). Shared human-robot proportional control of a dexterous myoelectric prosthesis. Nat. Mach. Intell..

[5] Geng W (2016). Gesture recognition by instantaneous surface EMG images. Sci. Rep..

[6] Guo W (2021). Long exposure convolutional memory network for accurate estimation of finger kinematics from surface electromyographic signals. J. Neural Eng..

[7] Mendez Guerra I, Barsakcioglu DY, Vujaklija I, Wetmore DZ, Farina D (2022). Far-field electric potentials provide access to the output from the spinal cord from wrist-mounted sensors. J. Neural Eng..

[8] Holobar A, Farina D (2021). Noninvasive neural interfacing with wearable muscle sensors: combining convolutive blind source separation methods and deep learning techniques for neural decoding. IEEE Signal Process. Mag..

[9] Bird JJ, Pritchard M, Fratini A, Ekart A, Faria DR (2021). Synthetic biological signals machine-generated by GPT-2 improve the classification of EEG and EMG through data augmentation. IEEE Robot. Autom. Lett..

[10] Tsinganos P, Cornelis B, Cornelis J, Jansen B, Skodras A (2020). Data augmentation of surface electromyography for hand gesture recognition. Sensors.

[11] Wang, F., Zhong, S.-h., Peng, J., Jiang, J. & Liu, Y. in *MultiMedia Modeling* (eds. Schoeffmann, K. et al.) 82–93 (Springer International Publishing, 2018).

[12] Zanini RA, Colombini EL (2020). Parkinson’s disease EMG data augmentation and simulation with DCGANs and style transfer. Sensors.

[13] Wen, S. et al. Rapid adaptation of brain-computer interfaces to new neuronal ensembles or participants via generative modelling. *Nat. Biomed. Eng.* https://www.nature.com/articles/s41551-021-00811-z#citeas (2021). 10.1038/s41551-021-00811-z PMC9114171 34795394

[14] Gootzen THJM, Stegeman DF, van Oosterom A (1991). Finite limb dimensions and finite muscle length in a model for the generation of electromyographic signals. Electroencephalogr. Clin. Neurophysiol. Evoked Potentials.

[15] Fuglevand AJ, Winter DA, Patla AE, Stashuk D (1992). Detection of motor unit action potentials with surface electrodes: influence of electrode size and spacing. Biol. Cybern..

[16] Stegeman DF, Linssen WH (1992). Muscle fiber action potential changes and surface EMG: a simulation study. J. Electromyogr. Kinesiol..

[17] Yue G, Fuglevand AJ, Nordstrom MA, Enoka RM (1995). Limitations of the surface electromyography technique for estimating motor unit synchronization. Biol. Cybern..

[18] Roeleveld K, Blok JH, Stegeman DF, Oosterom AV (1997). Volume conduction models for surface emg; confrontation with measurements. J. Electromyogr. Kinesiol..

[19] Schneider J, Silny J, Rau G (1991). Influence of tissue inhomogeneities on noninvasive muscle fiber conduction velocity measurements—investigated by physical and numerical modeling. IEEE Trans. Biomed. Eng..

[20] Botelho DP, Curran K, Lowery MM (2019). Anatomically accurate model of EMG during index finger flexion and abduction derived from diffusion tensor imaging. PLoS Comput. Biol..

[21] Carriou V, Boudaoud S, Laforet J, Ayachi FS (2016). Fast generation model of high density surface EMG signals in a cylindrical conductor volume. Comput. Biol. Med..

[22] Vecchio ADD (2020). Spinal motoneurons of the human newborn are highly synchronized during leg movements. Sci. Adv..

[23] Farina D, Mesin L, Martina S, Merletti R (2004). A surface EMG generation model with multilayer cylindrical description of the volume conductor. IEEE Trans. Biomed. Eng..

[24] Merletti R, Muceli S (2019). Tutorial. Surface EMG detection in space and time: best practices. J. Electromyogr. Kinesiol..

[25] Henneman E (1957). Relation between size of neurons and their susceptibility to discharge. Science.

[26] Girshick, R., Donahue, J., Darrell, T. & Malik, J. Rich feature hierarchies for accurate object detection and semantic segmentation. in *Proceedings of the IEEE Conference on Computer Vision and Pattern Recognition* 580–587 (2014).

[27] Clarke AK (2021). Deep learning for robust decomposition of high-density surface EMG signals. IEEE Trans. Biomed. Eng..

[28] Merletti, R. & Farina, D. *Surface Electromyography: Physiology, Engineering, and Applications* (John Wiley & Sons, Ltd, 2016).

[29] Dimitrov GV, Dimitrova NA (1998). Precise and fast calculation of the motor unit potentials detected by a point and rectangular plate electrode. Med. Eng. Phys..

[30] Farina D, Merletti R (2001). A novel approach for precise simulation of the EMG signal detected by surface electrodes. IEEE Trans. Biomed. Eng..

[31] Plonsey R (1977). Action potential sources and their volume conductor fields. Proc. IEEE.

[32] Plonsey R, Heppner DB (1967). Considerations of quasi-stationarity in electrophysiological systems. Bull. Math. Biophys..

[33] Heringa A, Stegeman DF, Uijen GJ, Weerd JPD (1982). Solution methods of electrical field problems in physiology. IEEE Trans. Biomed. Eng..

[34] Farina D, Mesin L, Martina S (2004). Advances in surface electromyographic signal simulation with analytical and numerical descriptions of the volume conductor. Med. Biol. Eng. Comput..

[35] Nicholson C, A. Freeman J (1975). Theory of current source density analysis and determination of conductivity tensor for anuran cerebellum. J. Neurophysiol..

[36] Pettersen, K. H., Lindén, H., Dale, A. M. & Einevoll, G. T. *Extracellular Spikes and Current-source Density* 92–135 (Cambridge University Press, 2010).

[37] Peter Knabner, L. A. *The Finite Element Method for the Poisson Equation* 46–91 (Springer New York, 2003).

[38] Vallaghé S, Papadopoulo T, Clerc M (2008). The adjoint method for general EEG and MEG sensor-based lead field equations. Phys. Med. Biol..

[39] Plonsey R (1974). The active fiber in a volume conductor. IEEE Trans. Biomed. Eng..

[40] Rosenfalck P (1969). Intra- and extracellular potential fields of active nerve and muscle fibres. A physico-mathematical analysis of different models. Acta Physiol. Scand. Suppl..

[41] Burke RE, Levine DN, Tsairis P, Zajac FE (1973). Physiological types and histochemical profiles in motor units of the cat gastrocnemius. J. Physiol..

[42] Schiaffino S, Reggiani C (2011). Fiber types in mammalian skeletal muscles. Physiol. Rev..

[43] Modenese L, Kohout J (2020). Automated generation of three-dimensional complex muscle geometries for use in personalised musculoskeletal models. Ann. Biomed. Eng..

[44] Keenan KG, Farina D, Merletti R, Enoka RM (2006). Influence of motor unit properties on the size of the simulated evoked surface EMG potential. Exp. Brain Res..

[45] Carriou, V., Laforet, J., Boudaoud, S. & Al Harrach, M. Realistic motor unit placement in a cylindrical HD-sEMG generation model. in *2016 38th Annual International Conference of the IEEE Engineering in Medicine and Biology Society (EMBC)* 1704–1707 (IEEE, 2016). 10.1109/EMBC.2016.7591044 28268655

[46] Fuglevand A, Winter DA, Patla AE (1993). Models of recruitment and rate coding organization in motor-unit pools. J. Neurophysiol..

[47] Ayachi FS, Boudaoud S, Marque CK (2014). Evaluation of muscle force classification using shape analysis of the sEMG probability density function: a simulation study. Med. Biol. Eng. Comput..

[48] Luca CJD, Hostage EC (2010). Relationship between firing rate and recruitment threshold of motoneurons in voluntary isometric contractions. J. Neurophysiol..

[49] Arabadzhiev TI, Dimitrov VG, Dimitrova NA, Dimitrov GV (2010). Influence of motor unit synchronization on amplitude characteristics of surface and intramuscularly recorded EMG signals. Eur. J. Appl. Physiol..

[50] Logg, A., Mardal, K. A. & Wells, G. N. *Automated Solution of Differential Equations by the Finite Element Method* Vol. 84. LNCSE of *Lecture Notes in Computational Science and Engineering* (Springer, 2012).

[51] Alnæs, M. et al. The FEniCS Project Version 1.5. *Arch. Numer. Softw.* **3**, 9–23 (2015).

[52] The CGAL Project. *CGAL User and Reference Manual*, 5.2.1 edn. https://doc.cgal.org/5.2.1/Manual/packages.html (CGAL Editorial Board, 2021).

[53] Negro F, Muceli S, Castronovo AM, Holobar A, Farina D (2016). Multi-channel intramuscular and surface EMG decomposition by convolutive blind source separation. J. Neural Eng..

[54] Cho, K. et al. Learning phrase representations using RNN encoder–decoder for statistical machine translation. in *Proceedings of the 2014 Conference on Empirical Methods in Natural Language Processing (EMNLP)* 1724–1734 (Association for Computational Linguistics, 2014).

[55] Loshchilov, I. & Hutter, F. Decoupled weight decay regularization. In *International Conference on Learning Representations (ICLR 2019)* (2019).

[56] Baxter J (1997). A Bayesian information theoretic model of learning to learn via multiple task sampling. Mach. Learn..

