# Supplementary material for: A myoelectric digital twin for fast and realistic modelling in deep learning
Source: Nat Commun. 2023 Mar 23;14:1600. doi: 10.1038/s41467-023-37238-w (PMC10036636; doi:10.1038/s41467-023-37238-w)
Supplement: Supplementary file 1 — Description of Additional Supplementary Files [file 41467_2023_37238_MOESM1_ESM.pdf]

Description of Additional Supplementary Files

File name: Supplementary Video 1
Description: Video tutorial of scripting a simulation pipeline in Python, showing the steps, parameters, functionality, and interactivity of the myoelectric digital twin tool.
