# Supplementary material for: A myoelectric digital twin for fast and realistic modelling in deep learning
Source: Nat Commun. 2023 Mar 23;14:1600. doi: 10.1038/s41467-023-37238-w (PMC10036636; doi:10.1038/s41467-023-37238-w)
Supplement: Supplementary file 3 — Reporting Summary [file 41467_2023_37238_MOESM3_ESM.pdf]

Corresponding author(s): Dario Farina
Last updated by author(s): Mar 2, 2023

Reporting Summary

Nature Portfolio wishes to improve the reproducibility of the work that we publish. This form provides structure for consistency and transparency in reporting. For further information on Nature Portfolio policies, see our Editorial Policies and the Editorial Policy Checklist.

Statistics

For all statistical analyses, confirm that the following items are present in the figure legend, table legend, main text, or Methods section.

n/a	Confirmed	
☐	☒	The exact sample size (*n*) for each experimental group/condition, given as a discrete number and unit of measurement
☒	☐	A statement on whether measurements were taken from distinct samples or whether the same sample was measured repeatedly
☐	☒	The statistical test(s) used AND whether they are one- or two-sided<br>*Only common tests should be described solely by name; describe more complex techniques in the Methods section.*
☐	☒	A description of all covariates tested
☒	☐	A description of any assumptions or corrections, such as tests of normality and adjustment for multiple comparisons
☐	☒	A full description of the statistical parameters including central tendency (e.g. means) or other basic estimates (e.g. regression coefficient) AND variation (e.g. standard deviation) or associated estimates of uncertainty (e.g. confidence intervals)
☐	☒	For null hypothesis testing, the test statistic (e.g. *F*, *t*, *r*) with confidence intervals, effect sizes, degrees of freedom and *P* value noted<br>*Give P values as exact values whenever suitable.*
☒	☐	For Bayesian analysis, information on the choice of priors and Markov chain Monte Carlo settings
☒	☐	For hierarchical and complex designs, identification of the appropriate level for tests and full reporting of outcomes
☒	☐	Estimates of effect sizes (e.g. Cohen's *d*, Pearson's *r*), indicating how they were calculated

Our web collection on statistics for biologists contains articles on many of the points above.

Software and code

Policy information about availability of computer code

Data collection	Data acquisition was carried out using public libraries for the Quattrocento amplifier (OT Bioelettronica) and force cells (Phidgets), and synchronized using a custom Matlab 2019b (The MathWorks, Inc) program as described in I. Mendez Guerra et al 2022 J. Neural Eng. 19 026031
Data analysis	The volume mesh of the simulator was generated from the surface meshes of the forearm tissues using the CGAL C++ library.<br>For the EMG simulation software (Myoelectric Digital Twin), the code is proprietary and is not publicly available. A development version of this software without specific version number was used in this study. To request the access to the simulation software, please contact kostiantyn.maksymenko@neurodec.ai<br>All machine learning was implemented using the PyTorch library in python.

For manuscripts utilizing custom algorithms or software that are central to the research but not yet described in published literature, software must be made available to editors and reviewers. We strongly encourage code deposition in a community repository (e.g. GitHub). See the Nature Portfolio guidelines for submitting code & software for further information.

Data

Policy information about availability of data

All manuscripts must include a data availability statement. This statement should provide the following information, where applicable:

- Accession codes, unique identifiers, or web links for publicly available datasets
- A description of any restrictions on data availability
- For clinical datasets or third party data, please ensure that the statement adheres to our policy

Surface geometry of muscles, bones, subcutaneous tissue and skin data used for arm modelling were taken from BodyParts3D, The Database Center for Life Science (http://lifesciencedb.jp/bp3d/).
The simulated MUAPs data that was used to train the neural network in the deep learning experiment have been deposited in the Science Data Bank database under DOI:10.57760/sciencedb.07548 (https://doi.org/10.57760/sciencedb.07548) and CC BY-NC-SA 4.0 licence.
The raw experimental data that was used in the deep learning experiment has also been deposited in the Science Data Bank database under DOI:10.57760/sciencedb.07586 (https://doi.org/10.57760/sciencedb.07586) and CC BY-NC 4.0 licence.

Human research participants

Policy information about studies involving human research participants and Sex and Gender in Research.

Reporting on sex and gender	The population of the experiment was comprised by 9 participants (4 females, 5 males - all self-reported). The study designed required a balanced gender (self-reported) population since data was pooled into a single dataset for the deep learning experiment due to the large number of needed data points.
Population characteristics	The population of the experiment was comprised by 9 participants (4 female, 5 male; ages: 23-31, all right-handed)<br>All participants were healthy and reported not pain, injury, or condition in the tested right upper limb.
Recruitment	Participants were recruited by advertising the experiment in the professional and personal networks. To minimise any potential experimenter bias, everyone that had an intact arm and did not experience a neuromotor nor a muscular disorder was accepted to participate in the study on a first come first served basis, as long as the population was gender-balanced.
Ethics oversight	Imperial College London ethics committee (JRCO: 18IC4685)

Note that full information on the approval of the study protocol must also be provided in the manuscript.

Field-specific reporting

Please select the one below that is the best fit for your research. If you are not sure, read the appropriate sections before making your selection.

[x] Life sciences [ ] Behavioural & social sciences [ ] Ecological, evolutionary & environmental sciences

For a reference copy of the document with all sections, see nature.com/documents/nr-reporting-summary-flat.pdf

Life sciences study design

All studies must disclose on these points even when the disclosure is negative.

Sample size	For the presented study, the sample size was determined based on previous studies exploring motor unit identification. Indeed, 9 participants was in line with the reported sample sizes of previous studies such as:<br>[1] Stachaczyk, Martyna, et al. "Toward universal neural interfaces for daily use: Decoding the neural drive to muscles generalises highly accurate finger task identification across humans." Ieee Access 8 (2020): 149025-149035.<br>[2] Kapelner, Tamas, et al. "Decoding motor unit activity from forearm muscles: perspectives for myoelectric control." IEEE Transactions on Neural Systems and Rehabilitation Engineering 26.1 (2017): 244-251.<br>[3] Martinez-Valdes, E., et al. "High-density surface electromyography provides reliable estimates of motor unit behavior." Clinical Neurophysiology 127.6 (2016): 2534-2541.
Data exclusions	For the purpose of training and testing the supervised decomposition pipeline, motor neuron activity was accepted if it was present for at least 80% of the contraction window (5 s in total)
Replication	Data collection from each participant was carried out independently. EMG signal preprocessing and decomposition initial parameters were the same for all participants. Once the network hyperparameters had been determined based on the training set (see below), each batch of the testing set was processed independently.
Randomization	The sEMG signal for each user was divided into a train and test set (first 4 seconds and last 1 second respectively) prior to any preprocessing or machine learning to prevent data leakage.

Blinding	During the experiment, participants were encouraged to perform constant isometric contractions to the best of their ability focusing on the force feedback only. Therefore, subjects were blind to their motor unit performance, which was only decomposed and analysed after data collection was finished.

Reporting for specific materials, systems and methods

We require information from authors about some types of materials, experimental systems and methods used in many studies. Here, indicate whether each material, system or method listed is relevant to your study. If you are not sure if a list item applies to your research, read the appropriate section before selecting a response.

Materials & experimental systems

n/a	Involved in the study
☒	☐ Antibodies
☒	☐ Eukaryotic cell lines
☒	☐ Palaeontology and archaeology
☒	☐ Animals and other organisms
☒	☐ Clinical data
☒	☐ Dual use research of concern

Methods

n/a	Involved in the study
☒	☐ ChIP-seq
☒	☐ Flow cytometry
☒	☐ MRI-based neuroimaging
